# Family history of hepatocellulcar carcinoma is not associated with its patients’ prognosis after hepatectomy

**DOI:** 10.1186/1477-7819-11-280

**Published:** 2013-10-18

**Authors:** Jia Huang, Yaojun Zhang, Meixian Chen, Junting Huang, Li Xu, Minshan Chen

**Affiliations:** 1 Department of Obstetrics and Gynaecology, First Affiliated Hospital of Sun Yat-sen University, 58 Zhongshan Road Second, Guangzhou 510089, China; 2 Department of Hepatobiliary Surgery, Sun Yat-sen University Cancer Center, 651 Dongfeng Road East, Guangzhou 510060, China; 3 State Key Laboratory of Oncology in South China, Sun Yat-sen University Cancer Center, Guangzhou 510060, China

**Keywords:** Hepatocellular carcinoma, Hepatectomy, TNM staging system, Family history, Prognosis

## Abstract

**Background:**

Family history of liver cancer is a major risk factor for hepatocellular carcinoma (HCC). In this study, we investigated the prognosis of patients with HCC with or without family history.

**Methods:**

Data for 1,313 patients who underwent hepatectomy as initial treatment for HCC between 2000 and 2008 at a tertiary cancer center hospital were retrieved from a prospective database. A positive family history was defined as a self-reported history of HCC in first-degree relatives. Clinicopathologic characteristics were compared by family history. Kaplan-Meier method and Cox proportional hazards regressions were applied for overall survival (OS) and disease-free survival (DFS).

**Results:**

Of 1,313 patients, 169 patients (12.9%) had first-degree relatives with a history of HCC. There were no significant differences between patients with or without family history in basic clinicopathologic characteristics. In either whole group or each stage according to the TNM staging system, first-degree family history was not associated with survival in all patients, hepatitis B virus-positive patients, as well as male patients. Multivariate analysis revealed that first-degree family history was not a prognostic factor, either for OS or DFS.

**Conclusion:**

A first-degree family history of HCC is not associated with its patients’ prognosis after hepatectomy.

## Background

Hepatocellular carcinoma (HCC) is the fifth most common cancer worldwide and the third most frequent cause of death due to cancer [1]. Although the majority of cases are still found in Asia and Africa, recent evidence has shown that both incidence and mortality rate of HCC are rising in North America and Europe [2-4]. More than 75% of cases worldwide and 85% of cases in developing countries have been attributed to hepatitis B virus (HBV) and hepatitis C virus (HCV), both of which increase the risk of HCC about 20-fold [5-8]. Other well-recognized risk factors for HCC include advanced age, male gender, heavy alcohol drinking, aflatoxin exposure, tobacco smoking, cirrhosis, and some rare monogenic syndromes (for example, hemocromatosis, alpha1-antitrypsin deficiency, and porphyria cutanea tarda) [6-8]. Recent studies [5-8] demonstrated that a family history of liver cancer increases HCC risk, independent of HBV/HCV infection status. The combination of family history of liver cancer and positive HBV/HCV serum markers is associated with an elevated HCC risk of >70-fold [8].

In the past decades the association between family history and cancer survival has been studied in various kinds of cancers, including colorectal cancer [9,10], gastric cancer [11], breast cancer [12], and so on. Most of them demonstrated that a positive family history was associated with improved survival. Since the effect of a positive family history of HCC on the survival remains unclear, we performed the present study to evaluate the effect of family history on the clinicopathologic characteristics and prognosis of patients who received hepatectomy as initial treatment for HCC in a Chinese population.

## Methods

### Ethics statement

The research was approved by the institutional review board (IRB) of Sun Yat-sen University Cancer Center, and written informed consent was obtained from each patient involved in the study.

### Patients

This is a retrospective study based on prospectively collected data at our hospital. Data on patients who underwent hepatectomy as initial treatment for HCC at the Department of Hepatobiliary Surgery, Sun Yat-Sen University Cancer Canter (Guangzhou, China) from 2000 to 2008 were retrieved from a prospective database. Only patients who met all of the following criteria were included: (1) no previous treatment for HCC before surgery; (2) histologic confirmation of HCC; (3) macroscopically completely resected, with a microscopically tumor-free margin proven by the pathology (R0 resection); (4) no lymph node or extrahepatic metastasis; (5) no history of other malignant disease; and (6) completed an interview about family history and health behavior. Since the current study aimed to analyze the prognostic factors for long-term survival, patients who died either perioperatively <30 days following surgery or during the original hospital stay if >30 days were excluded from this study.

All the preoperative parameters was recorded and evaluated as possible predictors of survivals including gender, age, HBV status, platelet count (PLT), prothrombin time (PT) alpha-fetoprotein (AFP), alanine transferase (ALT), total bilirubin level (TBIL), albumin (ALB), creatinine (Cr), liver function status, tumor size and number, and tumor thrombus. Liver functional reserve was also assessed using Child-Pugh classification. HBV positive was defined as HBsAg (+).

### Family history assessment

Family history of HCC was ascertained by self-reporting through an interview at the time of index case diagnosis. Cases with family history of HCC in a first-degree relative were recognized as those having at least one first-degree relative with HCC. First-degree relatives include parents, siblings, or offspring. Family history in second-degree relatives was not assessed in present analysis. No questions were asked about family size.

### Technique of hepatectomy

Hepatectomy was carried out by using the techniques we described previously [13]. In brief, hepatectomy was carried out under general anesthesia using a right subcostal incision with midline extension. Intraoperative ultrasonography was routinely performed to confirm resectability and to visualize major vascular structures. Pringle’s maneuver was routinely used with a clamp/unclamp time of 10/5 min. The liver parenchyma was divided with a clamp-crushing technique or ultrasonic dissector (CUSA) according to the surgeon’s preference. Anatomic resection was our preferred surgical method in hepatic resection for multiple nodules in one segment or in neighboring segments. For anatomic resection, the hepatic parenchyma was transected at the intersegmental plane as described by Couinaud. For multiple bilobar nodules, anatomic resection was preferred for the main tumor, while satellite nodules were resected non-anatomically with intent for a negative resection margin. When an inadequate liver remnant was suspected, non-anatomic resection was performed with intent for a negative resection margin. Hemostasis on the raw liver surface was achieved with suturing and fibrin glue.

### Follow-up

Patients were followed up every 3 months for the first 2 years, then every 6 months thereafter with physical examination, blood tests for AFP and liver function, and contrast abdominal computed tomography (CT). Chest radiography was done every 6 months to detect lung metastasis. When metastasis was suspected, CT chest, bone scintigraphy, positron emission tomography (PET), and biopsy if indicated were also performed to confirm metastasis and/or recurrence. The last follow-up date for patients still alive was in October 2012.

Causes of death and sites of recurrence were determined from death certificates, medical interviews, and radiological findings. Overall survival (OS) was defined as the interval between surgery and time of either death or last follow-up. Disease-free survival (DFS) was defined as the length of time after liver resection for HCC to detectable intrahepatic and/or extrahepatic recurrence. The treatment for recurrent tumors was determined by our multidisciplinary team (MDT) made up of surgeons, oncologists, radiologists, hepatologists, and pathologists.

### Statistical analyses

The statistical analyses were performed using the SPSS 13.0 statistical software (SPSS Company, Chicago, IL, USA). The two groups were compared using student’s t-test for continuous data and the Chi-square test for categorical data. The OS and DFS were calculated using the Kaplan-Meier method and compared using log-rank test. The prognostic factors in predicting OS and DFS were assessed by multivariate Cox proportional hazards regression analysis. All covariates that affected survival at the *P* <0.10 level of significance in univariate analysis were included in a multivariate Cox proportional hazards model. Results were given as mean ± SD. All statistical tests were two-sided, and a significant difference was considered when *P* <0.05.

## Results

### Baseline characteristics

A total of 1,427 patients with HCC who underwent hepatectomy as initial treatment were identified during the study period. Based on our inclusion criteria, 114 patients were excluded from this study, consisting of patients with lymph node metastasis (*n* = 11), positive resection margins (*n* = 77, 5.40%, 77/1,427), and those that died postoperatively (*n* = 26, mortality of 1.82%, 26/1,427).

Patients’ baseline characteristics were summarized in Table 1. A total number of 1,313 patients were included in the study, including 169 patients (12.9%, 169/1,313) had a family history of HCC. The demographic and clinicopathologic characteristics of patients with or without a family history of HCC were compared, and no significant difference was identified between these two groups.

**Table 1:** Clinicopathologic characteristics of the study population by family history

**Variables**	**All (*n*= 1,313)**		
	**Family history**		
	**Positive**	**Negative**	***P value***
	**(*n*= 169)**	**(*n*= 1144)**	
Age (>50/≤50 years)	54/115	510/634	0.101
Gender (M/F)	145/24	1002/142	0.514
ALT (u/L)	45.7 ± 38.7	51.5 ± 55.7	0.192
ALB (g/L)	42.0 ± 6.8	41.7 ± 4.5	0.497
TBIL (umol/L)	16.7 ± 9.7	18.7 ± 18.3	0.168
Cr (umol/L)	93.0 ± 20.7	92.2 ± 28.2	0.078
PT (s)	13.3 ± 1.2	13.3 ± 1.3	0.644
PLT count (×10^9^/L)	181.9 ±91.8	187.1 ± 76.9	0.837
AFP (≤400/>400 ng/mL)	98/71	637/507	0.573
HBV (positive/negative)	153/16	959/185	0.220
Liver cirrhosis (Yes/No)	149/20	940/204	0.302
Child Pugh (A/B)	167/2	1123/21	0.546
Tumor size (cm)	7.4 ± 4.2	7.3 ± 4.1	0.871
Tumor number (1/>1)	127/42	867/277	0.857
Major vascular invasion	19	144	0.621
Micro vascular invasion	19	125	0.902
Follow-up (months)	41.6 ± 32.8	44.7 ± 34.5	0.269
	(range 2.0–136.0)	(range 2.0–142.0)	

*AFP* alpha-fetoprotein; *ALT* alanine transferase; *ALB* serum albumin; *Cr* creatinine; *HBV* hepatitis B virus; *PLT* platelets; *PT* prothrombin time; *TBIL* total bilirubin.

### Effect of family history on survival

The median follow-up period was 44 months (range 2.5-73.5 months). The 1-, 3-, and 5-year OS for all patients were 79.2%, 55.4%, and 45.5%, respectively, and the median OS was 47 months. The 1-, 3-, and 5-year DFS was 52.6%, 36.1%, and 31.8%, respectively, and the median DFS was 15 months.

**Figure 1:**
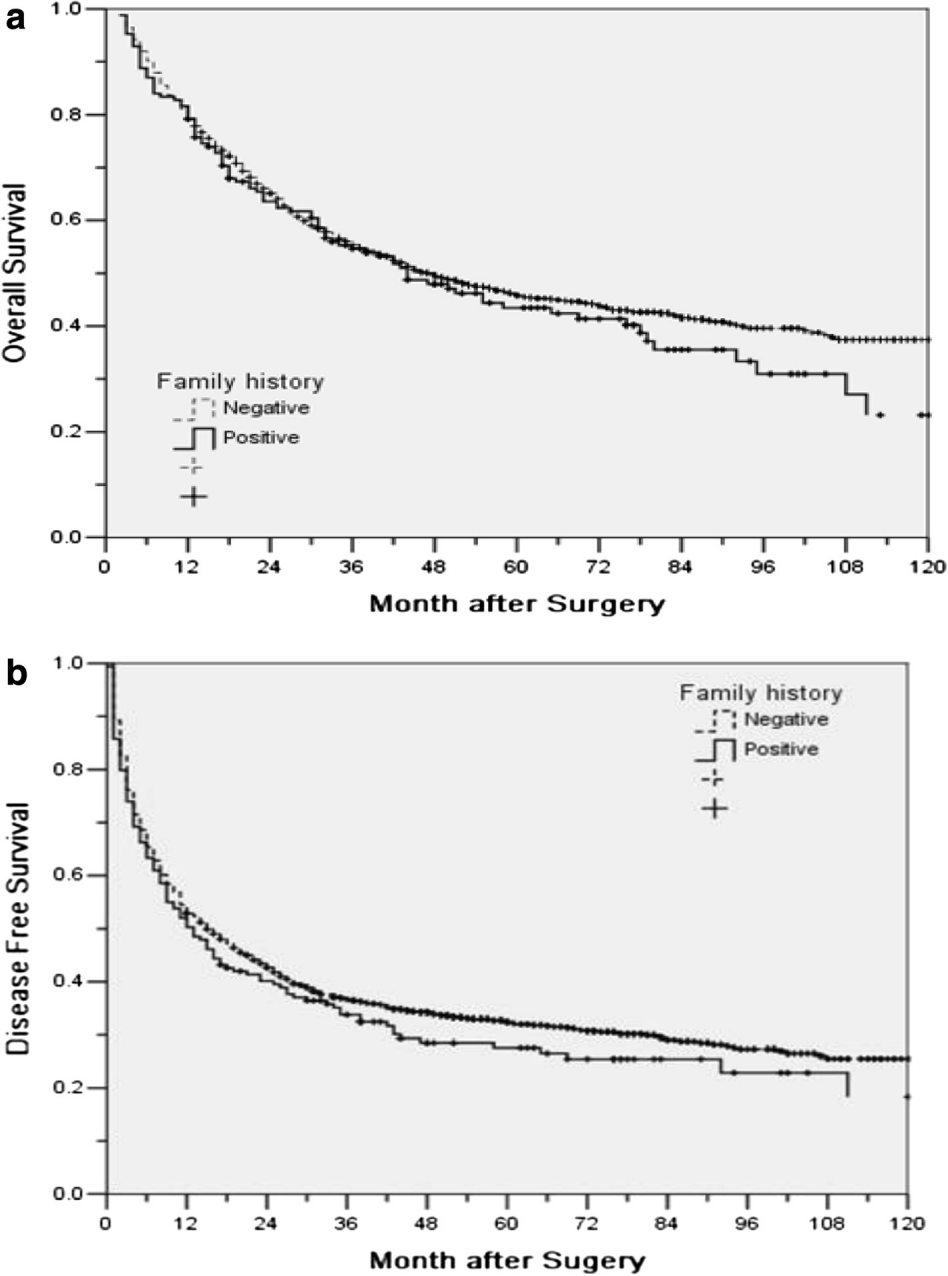
Overall (a) and disease-free survival (b) for patients with or without family history.

Table 2 summarized the OS and DFS of patients with or without a family history of HCC for all patients and subgroups according to TNM-7. For either OS or DFS, there was no significant difference in all patients nor in subgroups (Table 2, Figure 1).

**Table 2:** Overall and disease-free survival of the study population by family history and tumor stage

**TMN**	**Family history**	***n***	**Overall survival**				
			**Median survival (months)**	**1 year (%)**	**3 years (%)**	**5 years (%)**	***P*value**
All	Positive	169	43	79.3	54.7	43.5	0.511
	Negative	1,144	47	79.2	55.5	45.8	
I	Positive	87	79	87.4	71.0	58.9	0.282
	Negative	623	123	89.7	71.0	58.9	
II	Positive	21	32	81.0	44.2	36.8	0.441
	Negative	146	53	75.3	55.9	48.6	
III	Positive	61	22	67.7	34.7	24.8	0.550
	Negative	375	20	63.2	29.9	23.0	
**TMN**	**Family history**	***n***	**Disease-free survival**				
			**Median survival (month)**	**1 year (%)**	**3 years (%)**	**5 years (%)**	***P*value**
All	Positive	169	12	50.3	33.8	27.6	0.231
	Negative	1,144	14	52.9	36.7	32.4	
I	Positive	87	28	61.2	45.6	36.4	0.186
	Negative	623	35	66.8	49.2	44.5	
II	Positive	21	4	40.9	29.8	29.8	0.202
	Negative	146	12	49.0	35.8	32.2	
III	Positive	61	7	38.7	18.7	15.0	0.337
	Negative	375	5	31.5	16.2	12.2	

**Figure 2:**
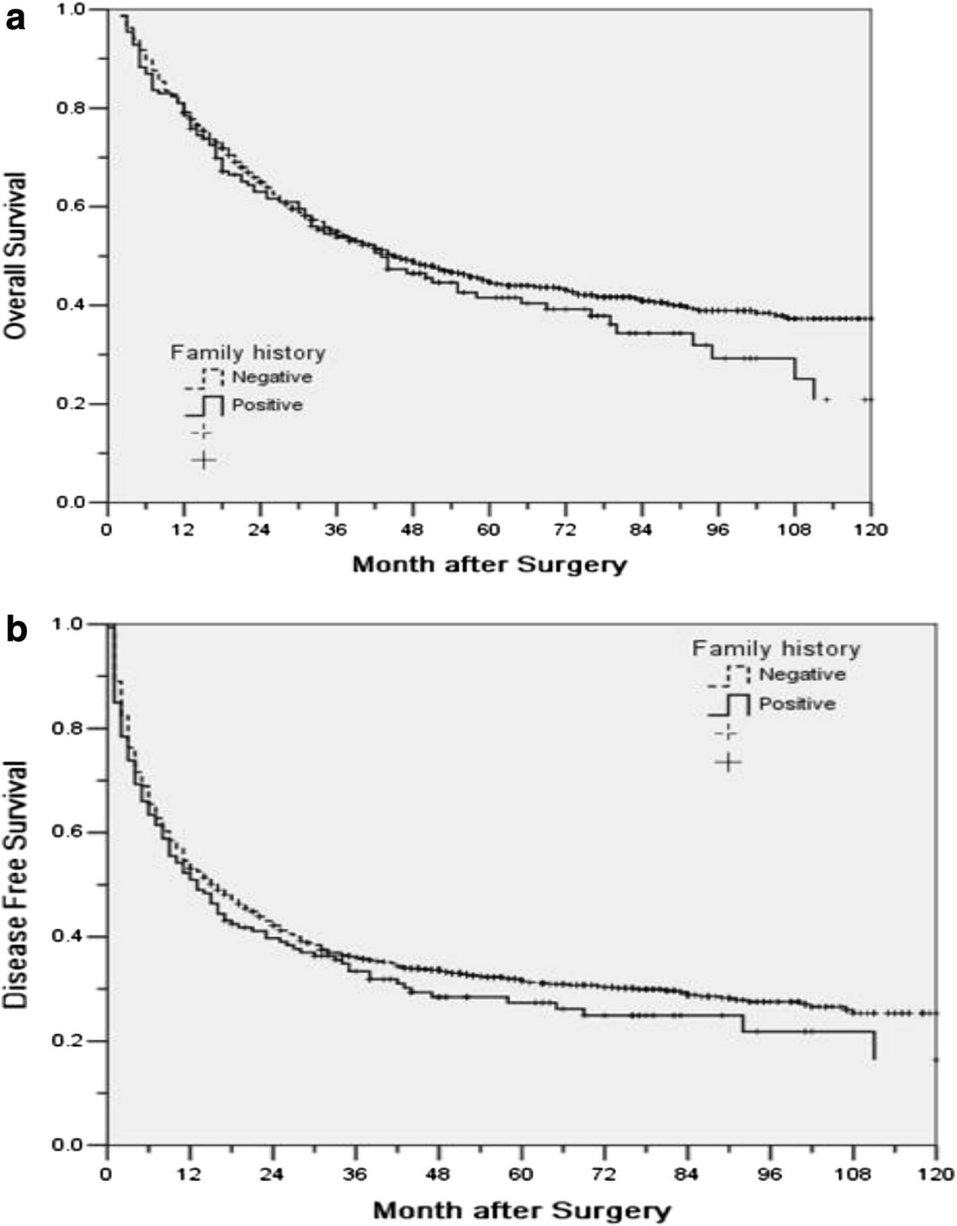
Overall (a) and disease-free survival (b) for HBV positive patients with or without family history.

Since the synergism between HBV and a family history of HCC was noted by several studies, the OS and DFS of HBV positive patients with or without a family history of HCC were compared in Table 3. Similarly, for either OS or DFS, there was no significant difference in all patients nor in subgroups (Table 3, Figure 2).

**Table 3:** Overall and disease-free survival of HBV positive patients by family history and tumor stage

**TMN**	**Family history**	***n***	**Overall survival**				
			**Median survival (month)**	**1 year (%)**	**3 years (%)**	**5 years (%)**	***P*value**
All	Positive	153	43	78.4	53.8	41.6	0.241
	Negative	959	46	79.1	54.9	44.7	
I	Positive	77	79	85.7	72.5	58.6	0.371
	Negative	529	105	90.2	71.0	57.5	
II	Positive	19	31	78.9	37.4	29.9	0.286
	Negative	115	48	73.0	53.8	46.6	
III	Positive	57	22	68.4	32.3	21.7	0.536
	Negative	315	19	62.9	27.8	22.1	
**TMN**	**Family history**	***n***	**Disease-free survival**				
			**Median survival (month)**	**1 year (%)**	**3 years (%)**	**5 years (%)**	***P*value**
All	Positive	153	13	51.0	33.4	27.4	0.246
	Negative	959	16	53.1	36.0	31.6	
I	Positive	77	28	62.3	45.1	36.6	0.174
	Negative	529	34	67.7	49.0	43.6	
II	Positive	19	4	36.9	23.0	23.0	0.179
	Negative	115	12	48.7	34.5	30.9	
III	Positive	57	8	40.4	20.4	16.3	0.189
	Negative	315	5	30.2	14.5	11.5	

**Figure 3:**
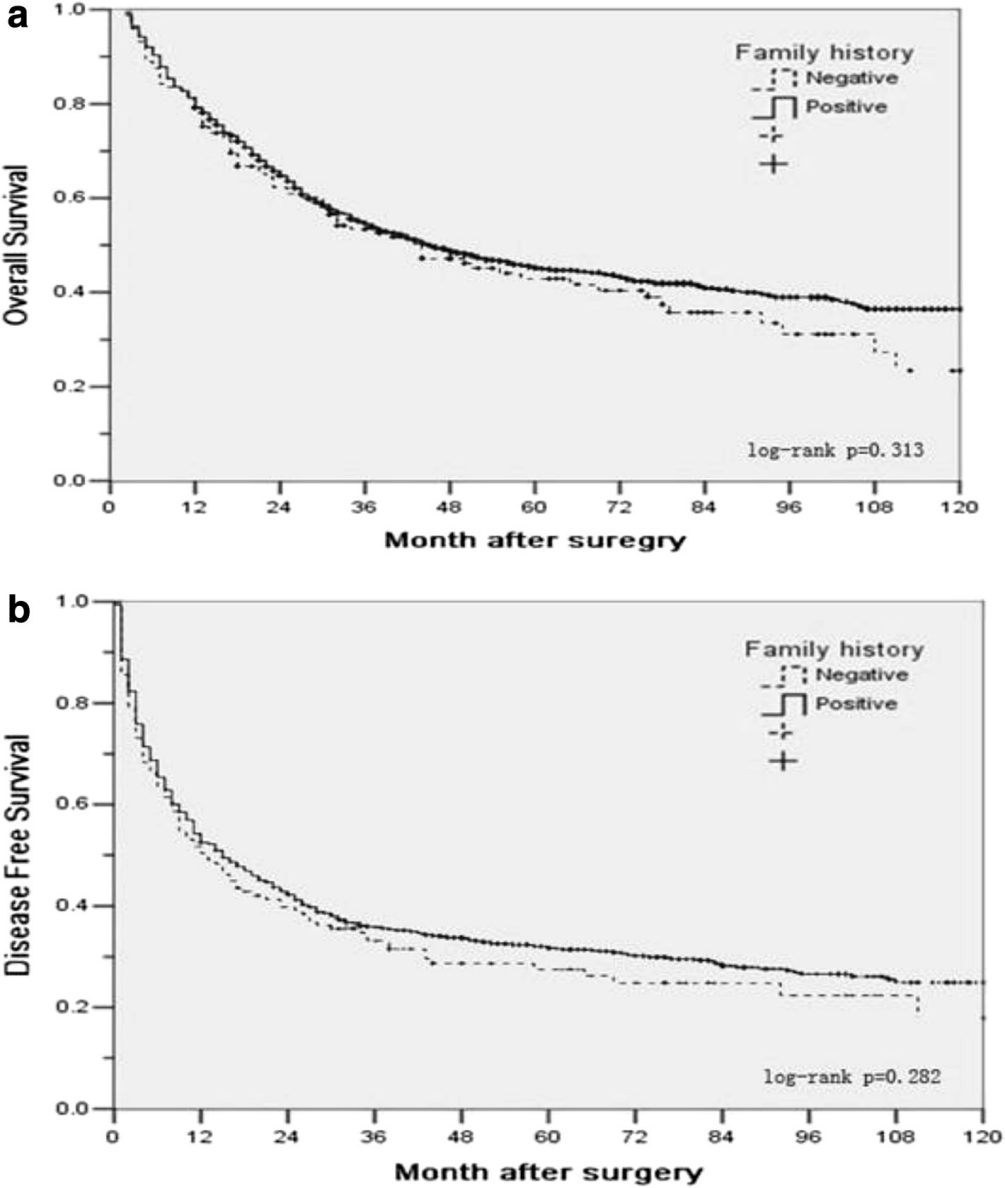
Overall (a) and disease-free survival (b) for male patients with or without family history.

It was also reported that a family history of HCC was only a risk factor in men but not women. The OS and DFS of male patients with or without a family history of HCC were compared in Figure 3. Similarly, there was no significant difference in either OS or in DFS (*P* = 0.313 and *P* = 0.282, respectively, Figure 3).

### Prognostic factors

All factors listed in Table 1 were included in univariate analysis and the covariates that affected survival at the *P* <0.10 level of significance in univariate analysis were included in a multivariate Cox proportional hazards model. Multivariate analysis showed that major vascular invasion (HR 2.624, 95% CI 2.149-3.203, *P* <0.001), tumor size (HR 1.670, 95% CI 1.405-1.986, *P* <0.001), tumor number (HR 1.569, 95% CI 1.333-1.846, *P* <0.001), PT (HR 1.424, 95% CI 1.217-1.666, *P* <0.001), ALB (HR 1.363, 95% CI 1.005-1.848, *P* = 0.046), PLT (HR 1.287, 95% CI 1.034-1.602, *P* = 0.024), and AFP (HR 1.179, 95% CI 1.011-1.375, *P* = 0.035) were significant prognostic factors for OS (Table 4).

**Table 4:** Multivariate analysis of prognostic factors for overall and disease-free survival

**Variables**	**Overall survival**		
	**HR**	**95.0% CI**	***P*value**
ALB (>35 /≤35 g/L)	1.363	1.005–1.848	0.046
PT (>15 /≤15 s)	1.424	1.217–1.666	<0.001
PLT count (>100/≤100 × 10^9^/L)	1.287	1.034–1.602	0.024
AFP (≤400/>400 ng/mL)	1.179	1.011–1.375	0.035
Tumor size (>5 /≤5 cm)	1.670	1.405–1.986	<0.001
Tumor number (1/>1)	1.569	1.333–1.846	<0.001
Major vascular invasion	2.624	2.149–3.203	<0.001
**Variables**	**Disease–free survival**		
	**HR**	**95.0% CI**	***P*value**
PT (>15 /≤15 s)	1.253	1.092–1.438	0.001
Tumor size (>5 /≤5 cm)	1.541	1.326–1.789	<0.001
Tumor number (1/>1)	1.617	1.397–1.872	<0.001
Major vascular invasion	2.456	2.036–2.962	<0.001
Micro vascular invasion	1.338	1.082–1.655	0.007

*AFP* alpha–fetoprotein; *ALB* serum albumin; *PLT* platelets; *PT* prothrombin time.

For DFS, multivariate analysis showed that major vascular invasion (HR 2.456, 95% CI 2.036-2.962, *P* <0.001), tumor number (HR 1.617, 95% CI 1.397-1.872, *P* <0.001), tumor size (HR 1.541, 95% CI 1.326-1.789, *P* <0.001), micro vascular invasion (HR 1.338, 95% CI 1.082-1.655, *P* = 0.007), and PT (HR 1.253, 95% CI 1.092-1.438, *P* = 0.001) were significant prognostic factors (Table 4).

Family history was not a significant prognostic factor for either OS or DFS.

## Discussion

Numerous studies [8,14-24] have demonstrated that a positive family history of liver cancer increases the risk of developing HCC. However, no previous studies have examined the influence of family history of liver cancer on subsequent outcomes in patients with established cancer. In this study, for the first time, we investigated the effect of family history on the clinicopathologic characteristics and prognosis of HCC. Our results showed that there was no significant difference in clinicopathologic characteristics and prognosis for patients with or without family history of HCC, either in all patients or in subgroups.

This study is based on family history as reported by patients, and only cases with a family history of HCC in first-degree relatives were included and analyzed. In the Connecticut Family Health Study [25], reports from first-degree relatives were more accurate than those from second-degree relatives, with positive predictive values between 78% and 80% for lung and breast cancer. Furthermore, several studies [5,8,14,24] have shown that only the family history of HCC in first-degree relatives increases the risk of developing HCC, but not second-degree relatives. Thus, we considered only first-degree relatives and second-degree relatives were not assessed in this study. We observed that 169 of the 1,313 patients (12.9%) had a family history of HCC in first-degree relatives. Our results were similar to those previous epidemiologic studies from Asian, USA, and European populations. Yu et al. [13] from China demonstrated that 17.5% of 553 patients reported having first-degree family members with HCC. Hassan et al. [8] from the USA observed that 6.1% of the 347 patients with HCC reported having first-degree family members with liver cancer. In a case-control study in Italy [24], 37 of 284 HCC patients (13.0%) reported having first-degree relatives with liver cancer.

Family history of the disease was found to be associated with improved survival in some kinds of cancers [9-12], including colon cancer, breast cancer, and so on. However there was some controversy in regards to gastric cancer. Han et al. [11] and Palli et al. [26] reported that a positive family history of gastric cancer in first-degree relatives was associated with a decreased risk of death and recurrence in patients after adjustments for known prognostic factors. However, a report from Japan [27] found that a family history negatively affected survival among gastric cancer patients. In Taiwan [28], the survival curve of patients with a positive family history was similar to that of patients without a family history. In our study, the impact of family history of HCC on both OS and DFS were analyzed in all patients and in subgroups according to TNM-7 stage. However, no significant difference was identified in patients with or without family history of HCC in either OS or DFS, in all patients or in subgroups.

The synergism between HBV/HCV and a family history of HCC was noted by several studies [5,8,14,24]. Turati et al. [24] reported that the combination of family history of liver cancer and HBV/HCV serum markers is associated to a >70-fold elevated HCC risk. The prognosis of HBV-positive patients with or without a family history was compared in our study. Similarly, there was no significant difference in either OS or DFS, in all patients or in subgroups. Only a few patients (9.5%, 16/169) with a family history were HBV-negative, the survival of HBV-negative patients with or without a family history cannot be analyzed in the present study.

Hassan et al. [8] observed that family history of liver cancer was only a risk factor for HCC in men but not in women. Similar results were also shown in a prospective longitudinal 90,000-person cohort study in China [14] and a case-control study from Italy [15]. But Turati et al. [24] and two Japanese studies [20,21] observed a non-significantly stronger association between HCC risk and family history of liver cancer for men. The OS and DFS of male patients with or without a family history of HCC were also compared in current study. However, no significant difference was identified either in OS or in DFS.

Several limitations of this study require commenting. First, as we relied on self-reported family history, misclassification of family history status may be possible. However, prior studies [25,29] have demonstrated such data to be reliable. Moreover, because the data on family history were collected at study baseline before treatment, any errors in recall would have attenuated rather than exaggerated a true association with patient outcome. Second, we did not collect information regarding number of siblings, and the likelihood of having a family history of the disease may vary according to the number of siblings at risk for the disease. However, it is unlikely that family size independently affects survival. Another limitation is the lack of information on family history of chronic hepatitis, cirrhosis, and other inherited diseases such as genetic hemochromatosis and alpha-1-antitrypsin deficiency, which may be related to HCC risk and prognosis [30-32]. Third, because our study was based on a database in a single tertiary cancer center hospital and all patients received hepatectomy as initial treatment, our results may not be generalizable to a larger population of patients with HCC, or patients treated with other methods including transcather artery chemoembolization, local ablation therapy, systemic chemotherapy, and so on. However, the rate of family history in this cohort is similar to the general population of patients with HCC.

Finally, we cannot completely exclude the possibility that patients with a family history may experience an earlier detection of malignancy. However, the effect of family history persisted after adjusting for other patient and disease characteristics associated with cancer recurrence or survival. Additionally, administration of treatment, therapies for recurrence disease, and follow-up care were reasonably uniform among all participants. Moreover, the association between family history and survival remained largely unchanged across the TNM stage as well as HBV status.

Studies suggested the importance of genetic contributions to the development of HCC. However, the relationship between family history and outcome is likely to be complex and may be influenced by a confluence of genetic and environmental factors [33-35]. We considered whether shared environmental or lifestyle factors might contribute to our findings. Further investigation to explore the relationship between family history and these factors is required.

## Conclusion

Our results showed that there was no significant difference in clinicopathologic characteristics and prognosis for patients with HCC after hepatectomy with or without family history of HCC, either in all patients or in subgroups.

## Competing interests

The authors declare that they have no competing interests.

## Authors’ contributions

JH and YZ carried out the molecular genetic studies, participated in the sequence alignment and drafted the manuscript. JH, YZ, MC, JH, and MC participated in the design of the study and performed the statistical analysis. LX and MC conceived of the study, and participated in its design and coordination and helped to draft the manuscript. All authors read and approved the final manuscript.

## References

[B1] Jemal A Bray F Center MM Ferlay J Ward E Forman D Global cancer statistics CA Cancer J Clin 2011 61 69 90 10.3322/caac.20107 21296855

[B2] Kew MC Hepatocellular carcinoma in developing countries: prevention, diagnosis and treatment World J Hepatol 2012 4 99 104 10.4254/wjh.v4.i3.99 22489262 PMC3321496

[B3] Lodato F Mazzella G Festi D Azzaroli F Colecchia A Roda E Hepatocellular carcinoma prevention: a worldwide emergence between the opulence of developed countries and the economic constraints of developing nations World J Gastroenterol 2006 12 7239 7249 17143937 10.3748/wjg.v12.i45.7239 PMC4087479

[B4] Bruix J Llovet JM Major achievements in hepatocellular carcinoma Lancet 2009 373 614 616 10.1016/S0140-6736(09)60381-0 19231618

[B5] Mazzanti R Gramantieri L Bolondi L Hepatocellular carcinoma: epidemiology and clinical aspects Mol Aspects Med 2008 29 130 143 10.1016/j.mam.2007.09.008 18061252

[B6] Stuver S Trichopoulos D Adami H-O, Hunter D, Trichpoulos D Cancer of the liver and biliary tract Cancer Epidemiology 2008 2 New York: Oxford Universiy Press 308 332

[B7] Marrero JA Fontana RJ Fu S Conjeevaram HS Su GL Lok AS Alcohol, tobacco and obesity are synergistic risk factors for hepatocellular carcinoma J Hepatol 2005 42 218 224 15664247 10.1016/j.jhep.2004.10.005

[B8] Hassan MM Spitz MR Thomas MB Curley SA Patt YZ Vauthey JN Glover KY Kaseb A Lozano RD El-Deeb AS Nguyen NT Wei SH Chan W Abbruzzese JL Li D The association of family history of liver cancer with hepatocellular carcinoma: a case–control study in the United States J Hepatol 2009 50 334 341 10.1016/j.jhep.2008.08.016 19070394 PMC2658718

[B9] Chan JA Meyerhardt JA Niedzwiecki D Hollis D Saltz LB Mayer RJ Thomas J Schaefer P Whittom R Hantel A Goldberg RM Warren RS Bertagnolli M Fuchs CS Association of family history with cancer recurrence and survival among patients with stage III colon cancer JAMA 2008 299 2515 2523 10.1001/jama.299.21.2515 18523220 PMC3616330

[B10] Zell JA Honda J Ziogas A Anton-Culver H Survival after colorectal cancer diagnosis is associated with colorectal cancer family history Cancer Epidemiol Biomarkers Prev 2008 17 3134 3140 10.1158/1055-9965.EPI-08-0587 18990755 PMC2739671

[B11] Han MA Oh MG Choi IJ Park SR Ryu KW Nam BH Cho SJ Kim CG Lee JH Kim YW Association of family history with cancer recurrence and survival in patients with gastric cancer J Clin Oncol 2012 30 701 708 10.1200/JCO.2011.35.3078 22271486

[B12] Thalib L Wedrén S Granath F Adami HO Rydh B Magnusson C Hall P Breast cancer prognosis in relation to family history of breast and ovarian cancer Br J Cancer 2004 90 1378 1381 10.1038/sj.bjc.6601694 15054458 PMC2409691

[B13] Chen MS Li JQ Zheng Y Guo RP Liang HH Zhang YQ Lin XJ Lau WY A prospective randomized trial comparing percutaneous local ablative therapy and partial hepatectomy for small hepatocellular carcinoma Ann Surg 2000 243 321 328 16495695 10.1097/01.sla.0000201480.65519.b8 PMC1448947

[B14] Yu MW Chang HC Liaw YF Lin SM Lee SD Liu CJ Chen PJ Hsiao TJ Lee PH Chen CJ Familial risk of hepatocellular carcinoma among chronic hepatitis B carriers and their relatives J Natl Cancer Inst 2000 92 1159 1164 10.1093/jnci/92.14.1159 10904089

[B15] Donato F Gelatti U Chiesa R Albertini A Bucella E Boffetta P Tagger A Ribero ML Portera G Fasola M Nardi G A case-control study on family history of liver cancer as a risk factor for hepatocellular carcinoma in North Italy Brescia HCC Study Cancer Causes Control 1999 10 417 421 10.1023/A:1008989103809 10530612

[B16] Huang YS Chern HD Wu JC Chao Y Huang YH Chang FY Lee SD Polymorphism of the N-acetyltransferase 2 gene, red meat intake, and the susceptibility of hepatocellular carcinoma Am J Gastroenterol 2003 98 1417 1422 10.1111/j.1572-0241.2003.07452.x 12818290

[B17] London WT Evans AA McGlynn K Buetow K An P Gao L Lustbader E Ross E Chen G Shen F Viral, host and environmental risk factors for hepatocellular carcinoma: a prospective study in Haimen City China Intervirol 1995 38 155 161 10.1159/000150426 8682610

[B18] Yu MW Yang SY Chiu YH Chiang YC Liaw YF Chen CJ A p53 genetic polymorphism as a modulator of hepatocellular carcinoma risk in relation to chronic liver disease, familial tendency, and cigarette smoking in hepatitis B carriers Hepatology 1999 29 697 702 10.1002/hep.510290330 10051470

[B19] Liu TT Fang Y Xiong H Chen TY Ni ZP Luo JF Zhao NQ Shen XZ A case-control study of the relationship between hepatitis B virus DNA level and risk of hepatocellular carcinoma in Qidong, China World J Gastroenterol 2008 14 3059 3063 10.3748/wjg.14.3059 18494059 PMC2712175

[B20] Tanaka K Hirohata T Takeshita S Hirohata I Koga S Sugimachi K Kanematsu T Ohryohji F Ishibashi H Hepatitis B virus, cigarette smoking and alcohol consumption in the development of hepatocellular carcinoma: a case-control study in Fukuoka, Japan Int J Cancer 1992 51 509 514 10.1002/ijc.2910510402 1318264

[B21] Tsukuma H Hiyama T Oshima A Sobue T Fujimoto I Kasugai H Kojima J Sasaki Y Imaoka S Horiuchi N Okuda S A case-control study of hepatocellular carcinoma in Osaka, Japan Int J Cancer 1990 45 231 236 10.1002/ijc.2910450205 2154409

[B22] Chen CJ Liang KY Chang AS Chang YC Lu SN Liaw YF Chang WY Sheen MC Lin TM Effects of hepatitis B virus, alcohol drinking, cigarette smoking and familial tendency on hepatocellular carcinoma Hepatology 1991 13 398 406 10.1002/hep.1840130303 1847891

[B23] Fernandez E La Vecchia C D'Avanzo B Negri E Franceschi S Family history and the risk of liver, gallbladder, and pancreatic cancer Cancer Epidemiol Biomarkers Prev 1994 3 209 212 8019368

[B24] Turati F Edefonti V Talamini R Ferraroni M Malvezzi M Bravi F Franceschi S Montella M Polesel J Zucchetto A La Vecchia C Negri E Decarli A Family history of liver cancer and hepatocellular carcinoma Hepatology 2012 55 1416 1425 10.1002/hep.24794 22095619

[B25] Mai PL Garceau AO Graubard BI Dunn M McNeel TS Gonsalves L Gail MH Greene MH Willis GB Wideroff L Confirmation of family cancer history reported in a population-based survey J Natl Cancer Inst 2011 103 788 797 10.1093/jnci/djr114 21562245 PMC3096799

[B26] Palli D Russo A Saieva C Salvini S Amorosi A Decarli A Dietary and familial determinants of 10-year survival among patients with gastric carcinoma Cancer 2000 89 1205 1213 10.1002/1097-0142(20000915)89:6<1205::AID-CNCR3>3.0.CO;2-5 11002214

[B27] Yatsuya H Toyoshima H Mizoue T Kondo T Tamakoshi K Hori Y Tokui N Hoshiyama Y Kikuchi S Sakata K Hayakawa N Tamakoshi A Ohno Y Yoshimura T Family history and the risk of stomach cancer death in Japan: Differences by age and gender Int J Cancer 2002 97 688 694 10.1002/ijc.10101 11807799

[B28] Lee WJ Hong RL Lai IR Chen CN Lee PH Huang MT Clinicopathologic characteristics and prognoses of gastric cancer in patients with a positive familial history of cancer J Clin Gastroenterol 2003 36 30 33 10.1097/00004836-200301000-00010 12488704

[B29] Kerber RA Slattery ML Comparison of self-reported and database-linked family history of cancer data in a case-control study Am J Epidemiol 1997 146 244 248 10.1093/oxfordjournals.aje.a009259 9247008

[B30] Bruix J Sherman M Management of hepatocellular carcinoma Hepatology 2005 42 1208 1236 10.1002/hep.20933 16250051

[B31] Bruix J Llovet JM Prognostic prediction and treatment strategy in hepatocellular carcinoma Hepatology 2002 35 519 524 10.1053/jhep.2002.32089 11870363

[B32] Shi M Guo RP Lin XJ Zhang YQ Chen MS Zhang CQ Wan Lau Y Li JQ Partial hepatectomy with wide versus narrow resection margin for solitary hepatocellular carcinoma: a prospective randomized trial Ann Surg 2007 245 36 43 10.1097/01.sla.0000231758.07868.71 17197963 PMC1867934

[B33] Volk ML Lok AS Is family history of liver cancer a risk factor for hepatocellular carcinoma? J Hepatol 2009 50 247 248 10.1016/j.jhep.2008.11.009 19070916

[B34] Zhu ZZ Cong WM Liu SF Xian ZH Wu WQ Wu MC Gao B Hou LF Zhu GS A p53 polymorphism modifies the risk of hepatocellular carcinoma among non-carriers but not carriers of chronic hepatitis B virus infection Cancer Lett 2005 229 77 83 10.1016/j.canlet.2005.04.014 15979781

[B35] Yu MW Yang YC Yang SY Chang HC Liaw YF Lin SM Liu CJ Lee SD Lin CL Chen PJ Lin SC Chen CJ Androgen receptor exon 1 CAG repeat length and risk of hepatocellular carcinoma in women Hepatology 2002 36 156 163 12085360 10.1053/jhep.2002.33897

